# Ist *Aloe vera* zur Prävention und Therapie der akuten radiogenen Proktitis geeignet?

**DOI:** 10.1007/s00066-021-01754-9

**Published:** 2021-03-04

**Authors:** Thomas Herrmann

**Affiliations:** TU Dresden, Fetscherstr. 74, 01307 Dresden, Deutschland

## Hintergrund

Die akute radiogene Proktitis stellt eine für die Patienten unangenehme Begleiterscheinung einer Strahlentherapie im Beckenbereich dar. Sicher sind zu ihrer Behandlung intrarektal applizierte Salben und Gels auch bei geringer Symptomatik oftmals genutzt und wohl auch nützlich. Die hier zu kommentierende doppelblind und randomisiert durchgeführte Studie sollte bei Patienten mit Beckenbestrahlungen die Frage beantworten, ob eine von den Patienten selbst mittels Rektumapplikator zweimal täglich intrarektal verabreichte Creme mit *Aloe-vera*-Anteilen von ca. 3 % eine stärkere Wirkung entfaltet bei der Linderung der akuten Proktitis als die Salbengrundlage allein ohne Wirkstoff.

## Patienten und Methodik

Bei insgesamt 42 Patienten (19 Verum versus 23 Placebo), die im Beckenbereich bestrahlt wurden (41–72 Gy über 6,5–7 Wochen bei Fraktionsdosen von 1,8 bis 2,0 Gy), wurden wöchentliche ärztliche Kontrollen durchgeführt. Dabei wurde anhand des Salbenverbrauchs die Zuverlässigkeit geprüft, mit der der Patient die intrarektalen Applikationen durchgeführt hatte, und der Patient hinsichtlich der Symptome einer Proktitis befragt. Als primäre Endpunkte dienten rektaler/abdominaler Schmerz, rektale Blutungen, Stuhlinkontinenz und imperativer Stuhldrang. Entsprechend wurden die Nebenwirkungen der Bestrahlung mit einer Skala von 0 bis 4 (schwere Störung) eingeteilt. Darüber hinaus wurden als sekundäre Endpunkte die RTOG-Kriterien für akute Toxizität sowie die Lebensqualitätsdaten mit entsprechenden Fragebögen erfasst.

Die Bestrahlungen wurden CT-geplant und das Rektum mit einem angepassten Block ausgeblendet. Neben Patienten mit Prostatakarzinomen wurden auch solche mit Harnblasen- und Zervixkarzinomen (ohne Angabe der jeweiligen Anzahl) in die Studie aufgenommen. Eine tabellarische Zusammenstellung dokumentiert in beiden Studienarmen eine vergleichbare Verteilung von Alter (61–63 Jahre), Geschlecht, Tumordosis, zusätzlichen Medikamenteneinnahmen und Komorbiditäten, z. B. Diabetes.

## Ergebnisse

65,2 % der Patienten im Placeboarm zeigten verschiedene Grade einer Proktitis. Im *Aloe*-Arm konnte diese Nebenwirkung nur bei 5 % der Patienten nachgewiesen werden. Mit Ausnahme von rektalen Schmerzen, Zystitis und Verstopfung zeigte sich bei allen sonstigen Nebenwirkungen eine sehr überzeugende Wirkung des Verumpräparats. Statistisch waren rektale Blutung, Diarrhö, imperativer Stuhldrang signifikant in der Verumgruppe verringert und damit auch die Lebensqualität bei diesen Patienten verbessert. Während die Symptome in der Verumgruppe über die gesamte Bestrahlungszeit nahezu konstant auf niedrigem Niveau blieben, nahmen sie in der Placebogruppe von Woche zu Woche zu. Bei einer biostatistischen Analyse aller untersuchten Parameter gab es neben den oben genannten Symptomen auch in den RTOG-Kriterien eine signifikante Verringerung der Nebenwirkungen. Allerdings verbesserten sich die pauschalen Angaben zu rektalem Schmerz, Proktitis allgemein und Zystitis nicht statistisch signifikant. Auch Entzündungszeichen (CRP) waren im *Aloe*-Arm geringer ausgeprägt.

Eine grafische Darstellung der Symptome zeigt über den sechswöchigen Bestrahlungsverlauf, dass alle Symptome auf dem von den Autoren gewählten Scoring-System mit den Graden 1–4 nur Veränderungen zwischen 0 und 1,4 aufwiesen. Auffällig ist dabei, dass in der *Aloe*-Gruppe die klinischen Symptome nie den Wert 0,2 überschritten bzw. bei den rektalen Schmerzen bereits nach der Hälfte der Bestrahlungsserie von 0,5 auf 0,2 zurückgingen.

## Schlussfolgerungen der Autoren

Die Autoren beurteilen die Ergebnisse ihrer Untersuchung als beweisend dafür, dass *Aloe vera* die Symptome der strahleninduzierten Proktitis, besonders die Diarrhö, Blutungen und imperativen Stuhldrang, so überzeugend verbessert, dass sich dieses Naturprodukt zur Prävention der radiogenen Proktitis empfiehlt.

## Kommentar

Die akute radiogene Proktitis stellt eine für den Patienten unangenehme Begleiterscheinung einer Strahlentherapie im Beckenbereich dar. Deshalb werden seit Jahren intensive planerische Bemühungen durchgeführt, um bei den Bestrahlungen die Rektumschleimhaut nur mit einer möglichst niedrigen Dosis zu belasten.

Das von den Autoren gewählte Scoring-System lehnt sich an das 4‑stufige System der RTOG-Kriterien [1] an, ohne jedoch auf die dort gemachten – auch klinisch beim Patienten leichter ermittelbaren – Symptome, wie beispielsweise Diarrhöfrequenz, abzustellen. Auf die besondere Schwierigkeit, zwischen radiogenen Veränderungen im Rektumbereich und im unteren Intestinum anhand der Stuhlfrequenzen nach Beckenbestrahlungen zu unterscheiden, wird bei allen Autoren, die sich mit der Skalierung dieser Veränderung beschäftigen, hingewiesen [2].

Bei den modernen strahlentherapeutischen Behandlungsverfahren [7] werden für die akute Proktitis der Grade 1 und 2 nach den CTC-Kriterien für Nebenwirkungen ca. 30 % angegeben. Die hier von den Autoren in der Einleitung ihres Artikels angegebene Häufigkeit von bis zu 75 % entspricht somit sicher nicht den heute in der modernen Strahlentherapie beobachteten Frequenzen. In der Arbeit von Barelkowski et al. [7] sind beispielsweise während der akuten Behandlungsperiode auch rektale Blutungen mit nur 6,8 % (bei 6 von 88 Patienten) angegeben.

Sicher ist die Verwendung von intrarektal applizierten Salben und Gels zur Behandlung der akuten Proktitis auch bei geringer Symptomatik nützlich. Allerdings kann es, sofern das, wie in der Studie beschrieben, dem ungeübten Patienten überlassen wird, im Handling mit dem Applikator bei zweimal täglicher Anwendung auch zu Mikrotraumen an der vulnerablen Rektumschleimhaut kommen, die dann Blutungen auslösen können. Offensichtlich ist aber bei der hier zu kommentierenden Arbeitsgruppe sowohl die Applikation von Verum als auch von Placebo bei den untersuchten Patienten erfolgreich, also unproblematisch gewesen. Insgesamt wurde nämlich nur eine geringe Symptomhäufigkeit beschrieben. Auf der von den Autoren gewählten Skalierung von 1 bis 4 zeigten die Patienten in der Placebogruppe maximal Symptome von 1,4 (Diarrhö), 1,0 (rektale Schmerzen) und 0,8 (imperativer Stuhldrang). Und die Blutungsneigung nahm während der sechswöchigen Bestrahlungsserie nur auf den Wert 0,4 zu. Hier könnte die möglicherweise unprofessionelle Verwendung des Rektalapplikators durch die Patienten die radiogenen Nebenwirkungen überlagert haben. Rektale Blutungen sind in der klinischen Praxis darüber hinaus eher Ausdruck einer späten als einer frühen Strahlennebenwirkung [3].

Die Aussagekraft der hier kommentierten Studie ist darüber hinaus erheblich eingeschränkt: erstens angesichts der geringen Patientenzahl in beiden Gruppen, zweitens wegen der nicht gut gewählten Spreizung des individuellen Scoring-Systems zur Erfassung der Symptome der radiogenen Proktitis und drittens wegen der ungenügend beschriebenen strahlentherapeutischen Technik. So sind wichtige Schlussfolgerungen bezogen auf die tatsächlich applizierte Dosis am Rektum nicht möglich.

Schließlich ist grundsätzlich die Anwendung des Naturprodukts *Aloe vera *bei der hier gewählten Indikation zu hinterfragen. Es gibt eine Vielzahl von Berichten, dass *Aloe* eine entzündungshemmende Wirkung besonders auf der Haut und dabei wiederum besonders bei Brandwunden entfalten kann. Neben der Anwendung von *Aloe *in sogenannten Nahrungsergänzungsmitteln werden Auszüge der Blätter der *Aloe vera* (Synonym: *Aloe barbadensis*) sowohl in Salben und Gels als auch wegen ihrer laxierenden Wirkung in sog. Gesundheitspflegemitteln angewandt. Für die orale Anwendung als Laxans werden als Gegenanzeige [4] neben Darmverschluss auch akut-entzündliche Erkrankungen des Darms angegeben. Dabei gelten die vom Bundesamt für Arzneimittel und Medizinprodukte (BfArM) bereits 1996 veröffentlichten Anwendungsbeschränkungen [5]: „Aus Untersuchungen an Zellkulturen, Tierversuchen, sowie der o. g. epidemiologischen Studie ergibt sich der begründete Verdacht, dass die in o. a. Arzneimitteln enthaltenen Anthranoid-Wirkstoffe genotoxisch und tumorigen wirken könnten. Es gilt also, die Patienten vor diesen unerwünschten Arzneimittelwirkungen zu schützen, die bei länger dauernder Anwendung und/oder hochdosiertem Mehrfachgebrauch auftreten können. Bei oraler Gabe ist deshalb die Anwendung auf 1–2 Wochen zu beschränken.“

Das Bundesinstitut für Risikobewertung (BfR) kommt 2017 in seinem ausführlichen Bericht [4] zusammenfassend zu der Schlussfolgerung: „Das BfR weist darauf hin, dass die IARC (Internationale Agentur für Krebsforschung) einen Extrakt aus ganzen Blättern von *Aloe barbadensis* (Synonym: *Aloe vera*) mit Bezug auf den Einsatz in Lebensmitteln (einschließlich Nahrungs-Ergänzungs-Mittel), kosmetischen Mitteln und als Laxans in Arzneimitteln bewertet hat. Es erfolgte eine Einstufung des Extraktes als ‚möglicherweise krebserregend beim Menschen‘ (Kategorie 2B, possibly carcinogenic to humans).“ Auch wenn in der hier kommentierten Studie *Aloe vera* in der Salbe in relativ niedriger Konzentration eingesetzt wird, ist doch u. a. durch die bei Bestrahlung vulnerable Rektumschleimhaut mit veränderten Resorptionsverhältnissen [6] eine unüberblickbare Aufnahme des Wirkstoffs nicht auszuschließen, zumal bei einer Bestrahlungsserie eine Anwendung auch über die empfohlenen zwei Wochen hinaus erfolgen müsste.

## Fazit

Zur Reduzierung der radiogenen Proktitis empfiehlt sich eine optimale Bestrahlungsplanung und -durchführung mit möglichst niedrigen Dosen an der Rektumschleimhaut und u. U. die Anwendung der in der klinischen Praxis seit Jahren bewährten Medikamente und Diäten. Meist lassen sich dadurch die Symptome der Patienten erfolgreich behandeln und verbessern.

Von einer Verwendung *Aloe*-haltiger, intrarektal zu applizierender Salben und Gels zur Prävention einer Proktitis ist angesichts der vorliegenden Literatur und der Empfehlungen des Bundesamts für Arzneimittel und Medizinprodukte abzuraten.

*Thomas Herrmann, Dresden*
